# A Compassion-Focused Ecological Momentary Intervention for Enhancing Resilience in Help-Seeking Youth: Uncontrolled Pilot Study

**DOI:** 10.2196/25650

**Published:** 2021-08-05

**Authors:** Christian Rauschenberg, Benjamin Boecking, Isabell Paetzold, Koen Schruers, Anita Schick, Thérèse van Amelsvoort, Ulrich Reininghaus

**Affiliations:** 1 Department of Public Mental Health Central Institute of Mental Health Medical Faculty Mannheim, Heidelberg University Mannheim Germany; 2 Department of Psychiatry and Neuropsychology School for Mental Health and Neuroscience Maastricht University Maastricht Netherlands; 3 Charité Universitätsmedizin Berlin Berlin Germany; 4 Mondriaan Mental Health Center Maastricht Netherlands; 5 ESRC Centre for Society and Mental Health King’s College London London United Kingdom; 6 Centre for Epidemiology and Public Health, Health Service and Population Research Department Institute of Psychiatry, Psychology & Neuroscience King’s College London London United Kingdom

**Keywords:** mental health, adolescent psychopathology, digital interventions, mobile health, self-compassion, ecological momentary assessment, mobile phone

## Abstract

**Background:**

Digital interventions offer new avenues for low-threshold prevention and treatment in young people. Ecological momentary interventions (EMIs) represent a powerful approach that allows for adaptive, real-time, and real-world delivery of intervention components in daily life by real-time processing of ecological momentary assessment (EMA) data. Compassion-focused interventions (CFIs) may be particularly amenable to translation into an EMI to strengthen emotional resilience and modify putative risk mechanisms, such as stress sensitivity, in the daily lives of young help-seeking individuals.

**Objective:**

This study aims to investigate the feasibility, safety, and initial therapeutic effects of a novel, accessible, transdiagnostic, ecological momentary CFI for improving emotional resilience to stress (*EMIcompass*).

**Methods:**

In this uncontrolled pilot study, help-seeking youth with psychotic, depressive, or anxiety symptoms were offered the EMIcompass intervention in addition to treatment as usual. The EMIcompass intervention consisted of a 3-week EMI (including enhancing, consolidating, and EMA-informed interactive tasks) administered through a mobile health app and three face-to-face sessions with a trained psychologist intended to provide guidance and training on the CFI exercises presented in the app (ie, training session, follow-up booster session, and review session).

**Results:**

In total, 10 individuals (mean age 20.3 years, SD 3.8; range 14-25) were included in the study. Most (8/10, 80%) participants were satisfied and reported a low burden of app usage. No adverse events were observed. In approximately one-third of all EMAs, individuals scored high on stress, negative affect, or threat anticipation during the intervention period, resulting in real-time, interactive delivery of the CFI intervention components in addition to weekly enhancing and daily consolidating tasks. Although the findings should be interpreted with caution because of the small sample size, reduced stress sensitivity, momentary negative affect, and psychotic experiences, along with increased positive affect, were found at postintervention and the 4-week follow-up. Furthermore, reductions in psychotic, anxiety, and depressive symptoms were found (*r*=0.30-0.65).

**Conclusions:**

Our findings provide evidence on the feasibility and safety of the EMIcompass intervention for help-seeking youth and lend initial support to beneficial effects on stress sensitivity and mental health outcomes. An exploratory randomized controlled trial is warranted to establish the feasibility and preliminary evidence of its efficacy.

## Introduction

### Background

Most mental disorders first emerge in adolescence and young adulthood (three-fourths by the age of 24 years [1]), with an estimated lifetime prevalence of approximately 50% of any mental disorder in young age groups [1-5]. Furthermore, the Global Burden of Disease study has reported that mental and substance use disorders in children and youth aged 10 to 24 years were the leading cause of overall disease burden in high-income countries [6-8]. Evidence further suggests that most mental disorders are continuous—phenomenologically and temporarily—and, in their early stages, are nonspecific in nature, often evolving into transdiagnostic phenotypes associated with a range of exit psychopathologies [9-16]. Consequently, clinical staging models as an adjunct to formal diagnoses have been introduced [17-19], highlighting the importance of transdiagnostic (indicated) prevention and early intervention [20-24].

Recent transformations in our understanding of the phenomenology, etiology, and early course of mental disorders have contributed to a move toward early detection and prevention [10-13,20,25-31]. Although conventional mental health services offer a range of therapeutic options, it has been widely documented that psychological help remains difficult to access, especially for young individuals in the early stages of mental health problems [21,22,32,33]. Furthermore, tailoring therapeutic options to specific needs and preferences of youth remains a challenge [32-36] and likely contributes to the problem that only a fraction of young people in need of help access any mental health service. Hence, young individuals often experience a long duration of untreated mental health problems, which has been identified as an important marker of poor course and outcome [32].

There is increasing interest in using digital tools to deliver mental health services [37], which may help extend access to and personalization of mental health care [38,39]. This shift has driven the development of novel mobile health (mHealth) interventions for various mental health problems [40-42], of which ecological momentary interventions (EMIs) [23,34,38,39,43], such as the Acceptance and Commitment Therapy in Daily Life [34-36,44], represent a very powerful approach. EMIs allow for adaptive, real-time, and real-world transfer of intervention components in individuals’ daily lives. Thus, EMIs provide a unique opportunity to deliver personalized, precision interventions tailored to what young individuals need in a given moment and context through interactive sampling in real time and the real world. They are based on fine-grained ecological momentary assessment (EMA) data acquired through cutting-edge digital technology [21,23,24,38,39,45,46]. More recently, some authors have started to use the term just-in-time adaptive interventions, which emphasize EMI’s capability of adapting the delivery of intervention components to person and context based on experience sampling or other, for example, sensing data [47,48].

One tangible prevention and early intervention strategy using digital tools is to identify and target transdiagnostic psychological mechanisms in daily life, which have been shown to be involved in the development of mental health problems [23,38]. In recent years, research using EMA—a structured diary technique, also known as experience sampling methodology [43]—has contributed to a better understanding of putative mechanisms likely to impact different stages and increase the intensity of mental health problems in individuals’ daily lives, in real time and outside the research laboratory [21-23,29,43,49,50]. To date, the psychological mechanism most widely studied in daily life is elevated stress sensitivity, characterized by more intense negative affective and psychotic experiences in response to minor stressors and routine daily hassles [22,24,29,43]. Previous studies have suggested that stress sensitivity is elevated in individuals with (1) higher familial or psychometric risk, (2) an ultra–high risk state for psychosis, (3) other early mental health problems, (4) first-episode psychosis, (5) severe and enduring psychosis, and (6) depressive disorders [21,22,24,28,50-58]. In addition, heightened interpersonal sensitivity and threat anticipation have previously been reported to represent further candidate mechanisms in individuals with ultra–high risk state for psychosis, paranoia, and psychotic disorders [24,29,30,59-62] and individuals with depression and anxiety [63-66]. These transdiagnostic mechanisms reflect candidate targets to be modified by EMIs [21,22,24,29].

Compassion-focused interventions (CFIs) are considered an important strand of transdiagnostic interventions for modifying emotion regulation systems [67,68]. CFIs are part of third-wave cognitive behavioral therapy (CBT) and previous meta-analytic evidence on third-wave CBT, including CFIs [69-73], suggest that these types of interventions may yield improvements in mental health outcomes of moderate-to-large effect size. CFIs have been successfully administered to and appraised positively by help-seeking individuals, including individuals with depression, anxiety, and psychosis [74-77]. Furthermore, CFIs have been shown to induce reductions in negative affect and paranoia in moments of high stress in previous research lab experimental work [78,79]. In addition, positive imagery, an important component of CFIs, has been found effective in reducing various mental health problems, including depression, anxiety, and psychosis [76,80,81] and increasing positive affect, optimism, and behavioral activation [79,82-84]. Thus, CFIs are particularly well placed to be administered as an EMI to strengthen emotional resilience and modify putative risk mechanisms of poor mental health in young individuals with psychological distress [72,78,85], including stress sensitivity and threat anticipation [21,22]. However, the use of conventional CFIs under real-world conditions remains limited [86].

As young individuals are *digital natives*, translating CFI components into an EMI administered through an mHealth app may be a particularly promising approach, offering entirely new avenues for low-threshold prevention and intervention in youth. EMIs are fundamentally translational as they directly build on evidence of underlying momentary mechanisms in daily life and translate these into the development and evaluation of novel digital interventions by targeting these mechanisms in real time and the real world, outside the research lab or clinic [23,39,43]. However, it remains to be established whether evidence on reductions in negative affect and paranoia in moments of high stress—observed in the research laboratory—and effects on other mental health outcomes can indeed be translated to real-world and real-time delivery of EMIs that harness CFI techniques, especially in young help-seeking individuals, where accessible, youth-friendly translation of prevention and early intervention principles reflects a particular challenge.

### This Study

The current study aims to establish the clinical feasibility, safety, and initial therapeutic effects of a novel, accessible, transdiagnostic, ecological momentary CFI for improving emotional resilience to stress (*EMIcompass*) in an uncontrolled phase 1 pilot study in help-seeking youth with psychotic, depressive, or anxiety symptoms. The EMIcompass intervention consisted of a 3-week EMI and three face-to-face sessions with a trained psychologist (ie, training session, follow-up booster session, and review session). Specifically, the intervention offered widely used CFI techniques (eg, compassionate and positive imagery, compassionate writing, and emotion as a wave). To facilitate the interactive, real-time, and real-world translation of the therapeutic content and techniques used in the initial training and booster sessions into individuals’ daily lives, the EMI was administered through an mHealth app on a smartphone. The EMI consisted of (1) enhancing tasks, (2) consolidating tasks, and (3) EMA-informed interactive tasks that aim at an ecological translation of CFI principles and techniques to daily life. Participants were required to complete one *enhancing task* per week, which allowed them to practice new compassion-focused exercises that were then extended throughout the study period. In addition, they were required to practice the learned CFI components once a day by completing the *consolidating tasks*. Each time an enhancing task was presented, the intervention components covered by consolidating tasks were expanded. Participants were also offered *interactive tasks* if they scored high on stress, negative affect, or threat anticipation in daily EMA. The face-to-face sessions were designed to provide guidance and training on the CFI exercises and how to use the app, background information on the strategies presented, and discussions of open questions and challenges participants encountered while using the app.

The primary objective of this study is to (1) assess the clinical feasibility of delivering the EMIcompass intervention to help-seeking youth based on successful recruitment, assessment of outcomes, compliance, satisfaction, and acceptability and safety by carefully documenting any serious adverse events throughout the study period. The secondary objectives were to examine (2) initial therapeutic effects of EMIcompass on reducing stress sensitivity, negative affect, and psychotic experiences, and increasing positive affect in daily life at the end of the 3-week intervention period (*postintervention*), and after a 4-week follow-up period (*follow-up*), along with (3) the initial therapeutic effects of EMIcompass on reducing threat anticipation, psychotic, depressive, and anxiety symptoms as well as general psychopathology.

## Methods

### Study Design

In an uncontrolled phase 1 pilot study, help-seeking individuals with psychotic, depressive, or anxiety symptoms aged between 14 and 25 years were referred to secondary mental health services in the Netherlands (ie, Mondriaan Mental Health Trust and Virenze Mental Health Care) and received the EMIcompass intervention in addition to treatment as usual. Data were collected before the intervention (*baseline*), at the end of the 3-week intervention period (*postintervention*), and after a 4-week follow-up period (*follow-up*). Close attention was paid to establishing the clinical feasibility (eg, pragmatic inclusion and exclusion criteria based on routine assessments) and safety (ie, documentation of any serious adverse events) of this study. Our recruitment strategy drew on our previous and ongoing work with youth [22,24,29,34-36,44] and guidance for pragmatic randomized controlled trials (RCTs) [87] and hence was geared to reflect the heterogeneity of the population commonly encountered in routine care.

### Sample

**Figure 1:**
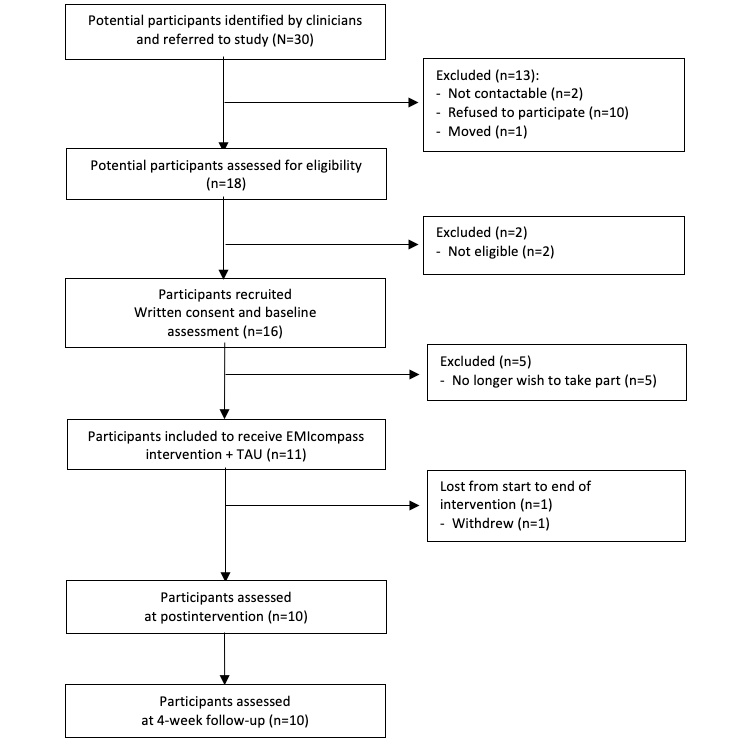
Study flowchart. TAU: treatment as usual.

We recruited young individuals with psychotic, depressive, and/or anxiety symptoms who sought help from two secondary mental services (ie, Mondriaan Mental Health Center and Virenze Mental Health Care). The inclusion and exclusion criteria were equivalent in principle across the two services but were purposefully selected to be pragmatic and hence based on routine assessments for screening, diagnosis, formulation, and outcome measurement, which differed between the two services (Textbox 1). This approach was adopted to ensure that the aim of establishing feasibility reflected the population actually encountered in clinical practice (rather than imposed by researchers) while keeping the assessment burden at a minimum. The study was approved by the Ethics Review Committee of Mondriaan Mental Health Center and the Ethics Review Committee of Psychology and Neuroscience, Maastricht University. A flowchart of the study is shown in Figure 1.

The prodromal questionnaire (PQ) [88,89], which has been reported to be a very good screening measure in routine mental health services [89,90], was used to screen for psychotic symptoms. In addition, the Brief Symptom Inventory (BSI) [91,92] was used to screen for anxiety, depressive, and psychotic symptoms, and the Symptom Questionnaire-48 [93] was used in addition to the PQ to screen for anxiety and depressive symptoms.

**Inclusion and exclusion criteria by participating mental health services.**

**Inclusion Criteria**

Mondriaan

- Aged between 18 and 25 years
- Prodromal questionnaire score of 6 or above
- Symptom questionnaire-48 score of 9 or above on the social phobia subscale, or score of 8 or above on the depression subscale, or score of 11 or above on the anxiety subscale
- Willingness to participate in the compassion-focused ecological momentary intervention
- Ability to give written informed consent independently, without help from others

Virenze

- Aged between 14 and 25 years
- Prodromal questionnaire score of 6 or above
- Brief Symptom Inventory *t* score of 63 or above
- Willingness to participate in the compassion-focused ecological momentary intervention
- Ability to give written informed consent independently, without help from others

**Exclusion Criteria**

- Insufficient command of Dutch, primary clinical diagnosis of alcohol or substance dependency, severe endocrine, cardiovascular, or organic brain disease

### The EMIcompass Intervention

#### Development of the Manual

The intervention was structured and manualized to ensure consistent delivery. The manual was based on widely used CFI techniques (eg, compassionate and positive imagery, compassionate writing, and emotion as a wave) and developed following a process of reviewing existing manuals and the extant CFI literature [67,68,73,74,78,80] through the team’s clinical experience of working with these approaches with clients and through consultation with local experts in CFI and the wider research team. The intervention was designed based on the principles of EMIs [23,34-36,39,43,44].

#### EMIcompass Intervention and Treatment as Usual

In this study, participants were offered the EMIcompass intervention in addition to treatment as usual, which included all the treatment they received before the start of the study (ie, good standard care delivered according to local and national guidelines by their general practitioner, psychiatrist, and other health care professionals), including CBT, third-wave CBT, dialectical behavior therapy, and other psychological interventions. The EMIcompass intervention consisted of three face-to-face sessions (one training session, one follow-up booster session, and one review session) given by a trained psychologist, who was supervised by an expert clinical psychologist in compassion-focused therapy, and a 3-week EMI administered through an mHealth app on a smartphone (PsyMate; Psymate BV). In addition, participants were offered on-demand email and/or phone contact during the intervention period.

At the beginning of the 3-week intervention period, an initial face-to-face training session was offered to participants. This session was fully manualized based on previous research that used CFIs [67,68,74,78,94]. The goal of the first session was to train individuals to cope with negative emotions by applying a personal, compassionate image that conveys compassion, care, and warmth to them based on the description of Gilbert [68], as applied by Lincoln et al [78]. This was followed by inducing negative emotions using in-sensu exposure to a personally relevant social situation that participants remember having experienced as distressing. This method has been safely applied to individuals with mental health problems [74,78] without any adverse consequences or health-related risks. Following the induction of negative emotions, participants were asked to practice a 5-minute application of the compassionate image they were trained in at the beginning of the session [67,68,78]. This step of actively using compassionate imagery after inducing negative emotions is considered essential for compassion-focused therapy to be efficacious in reducing stress sensitivity, threat anticipation, and psychotic, depressive, or anxiety symptoms in daily life [67,68]. Training the use of compassionate imagery was repeated and extended to imagery involving a *compassionate self* [68] and *emotion as a wave* [94] in the following booster session 2 weeks after the initial training session. In the review session at the end of the 3-week intervention period, the smartphone was returned, and progress and satisfaction with and acceptability of the intervention were reviewed and assessed.

To allow for interactive, real-time, and real-world translation of the therapeutic content and techniques of initial and booster sessions into individuals’ daily lives, participants were offered a 3-week EMI delivered through an mHealth app. During the 3-week intervention period, the smartphone prompted a signaling sound from the smartphone seven times per day on 6 consecutive days per week to reduce the burden associated with app usage. At each beep, participants were asked to complete a brief EMA on momentary stress, positive and negative affect, and threat anticipation in daily life (see the section on EMA measures used). The EMA was scheduled at random within set blocks of time. The EMI consisted of 3 different types of tasks (Table 1): participants were asked to complete one *enhancing task* per week, allowing them to practice new compassion-focused exercises, which were subsequently extended during the study period (eg, discovering their own compassionate self and experiencing emotions as a wave). In addition, they were asked to practice the learned CFI components once a day by completing the *consolidating tasks* at a predefined time. The components covered by consolidating tasks were extended each time an enhancing task was presented. Furthermore, *interactive tasks* were offered if participants scored high on stress, negative affect, or threat anticipation in the EMA (ie, scores higher than 4 on a 7-point Likert scale). As an essential element of compassion-focused therapy is the use of compassionate imagery in moments of high stress, negative affect, or threat anticipation, these interactive tasks are thought to reflect a core active component of the 3-week compassion-focused EMI.

**Table 1:** Components of the EMIcompass intervention.

	Week 1	Week 2	Week 3
Compassion-focused training sessions	Training session (compassionate image)	Booster session (day 11-15; compassionate self-training, “emotion as a wave”)	Review session (after day 20)
Enhancing tasks	Task 1 (day 3 or 4): compassionate self-validation	Task 2 (day 9 or 10): “emotion as a wave”	Task 3 (day 15 or 16): self-compassionate writing
Consolidating tasks	Compassionate self-validation (from day 5, following enhancing EMIa task 1)	Compassionate self-validation “Emotion as a wave” (from day 11, following enhancing EMI task 2)	Compassionate self-validation “Emotion as a wave” Self-compassionate writing (from day 17, following enhancing EMI task 3)
Interactive tasks	Compassionate image Compassionate self-validation (from day 5, following enhancing EMI task 1)	Compassionate image Compassionate self-validation “Emotion as a wave” (from day 11, following enhancing EMI task 2)	Compassionate image Compassionate self-validation “Emotion as a wave” Self-compassionate writing (from day 17, following enhancing EMI task 3)

^a^EMI: ecological momentary intervention.

### Measures

#### Sociodemographic Characteristics

A sociodemographic schedule was used to assess age, gender, occupation, and level of education.

#### Clinical Feasibility and Safety

Feasibility was assessed based on successful recruitment, assessment of outcomes, compliance with the manual, satisfaction, and acceptability. For some of the feasibility domains, a debriefing scale was used. The reasons participants declined to participate in the study were carefully recorded, and the completeness of outcomes at each time point was documented. Acceptability was assessed in the review session of the EMIcompass intervention together with the trained psychologist by asking participants to complete a feedback form about the EMI tasks and sessions and rate the extent to which they felt they benefited from and were satisfied with the intervention [74,78]. In addition, the trained psychologist asked participants in the review session to report whether they perceived the face-to-face sessions, compassion-focused exercises, and EMI tasks as helpful. App usability was assessed by asking participants to rate the readability of the text shown on the screen, any difficulties in operating the app or technical problems, the clarity of provided instructions, and whether the app was perceived as burdensome. All items were rated on a 7-point Likert scale ranging from *not at all* (rating of 1) to *moderate* (rating of 4) and *very* (rating of 7), which were subsequently grouped into three categories of *not* (rating of 3 or lower), *moderate* (rating of 4 or 5), and *very* (rating of 6 or 7) for the sake of interpretability of findings (given small numbers in each cell). Safety was assessed by carefully documenting any serious adverse events throughout the entire study period and the potential negative effects of app usage on mental health in participants.

#### Stress Sensitivity, Negative and Positive Affect, and Psychotic Experiences in Daily Life

EMA was used to assess stress sensitivity, negative and positive affect, psychotic experiences, and threat anticipation in daily life. For this, the same app was used as for the EMIcompass intervention (PsyMate), and assessments were completed at baseline, postintervention, and 4-week follow-up for 6 consecutive days, following the protocol from previous EMA studies [22,24,29,46,49]. Stress was operationalized as minor disturbances and distinctive unpleasant events, activities, and social situations that occur in the flow of daily life. Event-related stress was measured with an item asking participants to rate the most important event that had happened since the last beep on a 7-point Likert scale ranging from *very unpleasant* (rating of −3) to *very pleasant* (rating of 3) [54]. The item was recoded, such as higher ratings indicated higher levels of stress (with ratings of −3 coded as 7 and ratings of 3 coded as 1). Activity-related stress was measured by asking participants first to specify their current activity (eg, resting and watching TV), which was followed by asking them to rate the pleasantness of this activity on a 7-point Likert scale (1=*very unpleasant*; 7=*very pleasant*). Social stress was measured by asking participants to specify categorically with whom they were spending time (eg, nobody, partner, or family) and appraise the current social context using the items “I find being with these people pleasant” (reversed), “I feel accepted” (reversed), and “I feel excluded (if with someone)” or “I find it pleasant to be alone” (reversed) and “I would prefer to have company” (if alone) ranging from *not at all* (rating of 1) to *very much* (rating of 7). The good concurrent validity of these EMA stress measures has been reported [54,55]. Furthermore, a composite stress score was calculated using the mean score of all seven stress items [21,95]. Negative affect was assessed using five items asking participants to rate the extent to which they felt anxious, down, insecure, uncomfortable, and guilty at each entry point [54]. Positive affect was assessed by asking participants to rate the extent to which they felt cheerful and relaxed, all rated on a 7-point Likert scale ranging from *not at all* (rating of 1) to *very much* (rating of 7) [54,55,96]. Psychotic experiences were assessed using seven items (“I see things that aren’t really there,” “I hear things that aren’t really there,” “I feel suspicious/paranoid,” “I feel unreal,” “My thoughts are inﬂuenced by other,” “I can’t get these thoughts out of my head,” and “I feel like I am losing control”) rated on a 7-point Likert scale ranging from 1 (*not at all*) to 7 (*very much*) [55,96]. Threat anticipation was assessed by asking participants to think of what might happen in the next few hours and rate the item “I think that something unpleasant will happen” on a 7-point Likert scale (ranging from 1=*not at all* to 7=*very much*) [24,29]. Negative and positive affect, psychotic experiences, and threat anticipation scores were assessed by computing the mean scores. In line with earlier studies [22,24,29,46,49], items on stress, negative affect, and psychotic experiences were used as a proxy for individuals’ stress sensitivity in daily life by modeling the association between stress and (1) negative affect and (2) psychotic experiences. Thus, we conceptualized stress sensitivity in daily life as individuals’ affective and psychotic reactivity to minor daily stressors.

#### Psychotic, Depressive, and Anxiety Symptoms and General Psychopathology

We used non-EMA outcome measures to assess psychotic, depressive, and anxiety symptoms and general psychopathology. First, the BSI was used to assess depressive and anxiety symptoms (based on the respective BSI subscales) and general psychopathology by computing the Global Severity Index (based on 53 BSI items). Participants rated each item on a 5-point scale ranging from 0 (*not at all*) to 4 (*extremely*) [91,92]. Second, the Green et al, Paranoid Thoughts Scale, a reliable and valid scale, was used to assess psychosis [97]. The Green et al, Paranoid Thoughts Scale was modified to ask participants about paranoid ideation during the past week rather than the past month, given that the intervention period was only 3 weeks. A total score was computed using all 32 items (both with a 5-point scale: 1=*not at all*, 3=*somewhat*, and 5=*totally*). Third, the threat anticipation measure [98] was used to measure threat anticipation by asking participants to estimate the future likelihood of a list of threatening, neutral, and positive events happening to themselves and other people [62,98,99]. Items for threatening and neutral events were used to compute the total scores. Each event was rated separately for the likelihood that it will happen to oneself and another person on a 7-point scale (1=*not at all*; 7=*very likely*), resulting in four total sum scores (ie, threat anticipation-self, threat anticipation-other, neutral anticipation-self, and neutral anticipation-other), where higher scores indicate higher probability estimates. Finally, the PQ [88,89] was used to assess the presence of prodromal and attenuated psychotic symptoms (ie, positive symptoms, disorganized symptoms, negative symptoms, and general symptoms). This measure consists of 16 items that assess the presence of psychotic symptoms (0=false and 1=true), which were used to compute a total score (range 0-16). Good psychometric properties have been reported for these measures [88,97,98,100,101].

### Statistical Analysis

STATA 15.1 (StataCorp) was used to analyze the data. First, descriptive statistics were used, and CIs were constructed, as appropriate, to summarize the findings on feasibility and safety. Second, as EMA data have a multilevel structure, such that multiple observations (level 1) are nested within subjects (level 2), linear mixed models were used to control for within-subject clustering of multiple observations using the *mixed* command in STATA. Thus, to examine the effects of the EMIcompass intervention on reducing stress sensitivity, EMA stress variables and time points were included as independent variables and negative affect and psychotic experiences as the outcome variable in linear mixed models, which were fitted separately for each outcome variable. We then added two-way interaction terms for stress×time and used likelihood ratio tests (*lrtest* command) to evaluate improvement in model fit and the *lincom* command to compute linear combinations of coefficients to test our hypotheses on whether stress sensitivity was reduced at postintervention and the 4-week follow-up. We standardized continuous ESM (experience sampling method) variables (mean 0, SD 1) to interpret significant interaction terms. Family-wise error-corrected *P* values were computed to control for multiple testing by multiplying the unadjusted *P* values of the two-way interaction effects by the total number of tests (N=4) for each outcome. Third, to examine the effects of the EMIcompass intervention on other EMA outcome measures, time points were included as independent variables and negative affect, positive affect, psychotic experiences, and threat anticipation as the outcome variable in separate linear mixed models. All models were controlled for potential confounders (ie, age, gender, and level of education). Finally, we used Wilcoxon signed-rank tests to examine the effects of EMIcompass on non-EMA outcome measures of threat anticipation, psychotic, depressive, and anxiety symptoms and general psychopathology at postintervention and 4-week follow-up. The resulting z scores were used to calculate the effect sizes displayed in *r* as described by Rosenthal and DiMatteo [102].

## Results

### Sociodemographic and Clinical Characteristics

A flowchart of the study is shown in Figure 1 and basic sample characteristics in Table 2. In total, 30 potential participants aged between 14 and 25 years were referred to the study by clinicians from the two participating mental health services. Of these, 16 provided written informed consent and were eligible, of whom 11 completed the baseline assessment and were included in the EMIcompass intervention. A participant was lost during the 3-week intervention period, whereas 10 participants (mean age 20.3 years, SD 3.8; range 14-24) completed the EMIcompass intervention and both postintervention and 4-week follow-up assessments. Most participants were women (7/10, 70%) and were currently at school/university (6/10, 60%). Half of the participants had a clinical diagnosis of major depressive disorder (5/10, 50%) and met the criteria for a comorbid mental health condition. Most participants were of White Dutch ethnicity, and some reported having used cannabis during the previous 12 months (3/10, 30%).

**Table 2:** Basic sample characteristics of service users (N=10).

Characteristic		Value
Age (years), mean (SD; range)		20.3 (3.8; 14-25)
**Sex, n (%)**		
	Female	7 (70)
	Male	3(30)
**Ethnicity, n (%)**		
	White Dutch	6 (60)
	Other	1 (10)
	Missing value	3 (30)
**Level of education, n (%)^a^**		
	School	2 (20)
	Further	4 (40)
	Higher	4 (40)
**Occupation, n (%)**		
	School or education	6 (60)
	Employed (full- or part-time)	3 (30)
	Unstructured activities	1 (10)
**Cannabis use^b^, n (%)**		
	12 months	3 (30)
	Lifetime	4 (40)
**DSM-IV^c^ diagnosis, n (%)**		
	Major depressive disorder	5 (50)
	Attention-deficit/hyperactivity disorder	1 (10)
	Reactive attachment disorder	2 (20)
	None	2 (20)
	Comorbid condition^d^	5 (50)

^a^Categories defined as school (elementary school), further (voorbereidend middelbaar beroepsonderwijs [VMBO]; hoger algemeen voortgezet onderwijs [HAVO], and voorbereidend wetenschappelijk onderwijs [VWO]), and higher (hoger beroepsonderwijs [HBO], and wetenschappelijk onderwijs [WO]) of the Dutch educational system.

^b^On the basis of Composite International Diagnostic Interview section of Illegal Substance Use and defined as having used cannabis more than five times on its own initiative during the previous 12 months or lifetime.

^c^DSM-IV: Diagnostic and Statistical Manual of Mental Disorders, Fourth Edition.

^d^Consisting of the following diagnostic categories: panic disorder, attention-deficit/hyperactivity disorder, intermittent explosive disorder, borderline personality disorder, and parent-child relational problem.

### Clinical Feasibility and Safety

The clinical feasibility and safety findings are shown in Table 3. Almost all individuals (9/10, 90%) reported that participating in the study did not interfere with their daily activities. Most individuals reported being very (40%-50%) or moderately satisfied (40%-50%) with tasks delivered through the EMIcompass app and moderately (20%-30%) or very (60%) satisfied across face-to-face sessions. Most participants were also very (5/10, 50%) or moderately (2/10, 20%) successful in imagining a compassionate image. Some individuals reported that the intervention positively influenced social contacts (3/10, 30%; ratings of *moderate* and *very* combined) and levels of activity (4/10, 40%). All individuals were very satisfied with the face-to-face contact sessions and felt trained psychologists understood them. Although all participants reported that they were able to follow the instructions shown on the screen, observer ratings by trained psychologists, who also delivered the face-to-face sessions, indicated that some individuals might have had problems with this (1/10, 10% in session 1 and 2/20, 20% in session 3). Findings on app usability were satisfactory, and the burden associated with app usage was perceived to be low or very low across all time points (70%-90%), although some individuals (3/10, 30%) found the number of signals per day to be moderately burdensome. In addition, some individuals perceived the items used in the PsyMate app as difficult or unclear (2/10, 20%). No severe adverse events were observed during the study period.

In-app usage data during the intervention period suggest high completion rates of EMA assessments. Specifically, the EMIcompass app triggered 1260 signals asking participants to complete brief EMA assessments (126 for each person). Of these 1260 signals, individuals reacted to 467 (37.06%), although high variability between individuals was found (range 214/1260, 16.9% to 844/1260, 66.9%). Individuals scored high on stress, negative affect, or threat anticipation in 32.1% (150/467) of EMA assessments, resulting in real-time delivery of CFI intervention components in approximately 1 out of 3 of all completed EMA assessments. When considering the assessment of outcomes at baseline, postintervention, and follow-up, we found satisfactory compliance rates (no missing data for outcome measures filled in person and at least 18/60, 30% of all EMA assessments). Thus, when combining self-reports and in-app usage data, assessing outcomes and compliance with the manual was considered satisfactory. Furthermore, the conversion rate of recruitment was 3:1 (ie, from identified to included individuals; Figure 1), which is in line with previous research and considered successful recruitment.

**Table 3:** Findings on safety, feasibility, and app usability of the EMIcompass intervention.

		Ratings^a^		
		Very	Moderate	Not
**Safety and feasibility, n (%)**				
	Interference of study participation with daily activities	0 (0)	1 (10)	9 (90)
	Satisfaction with face-to-face sessions	6 (60)	2 (20)	2 (20)
	Session 1: compassionate image; inducing negative emotions	6 (60)	3 (30)	1 (10)
	Session 2: compassionate self; emotion as a wave	6 (60)	3 (30)	1 (10)
	Session 3: review session	6 (60)	3 (30)	1 (10)
**Satisfaction with tasks, n (%)**				
	Task 1: compassionate self-validation	4 (40)	5 (50)	1 (10)
	Task 2: emotion as a wave	5 (50)	3 (30)	2 (20)
	Task 3: self-compassionate writing	5 (50)	3 (30)	2 (20)
	Self-reported success in making a compassionate image	5 (50)	3 (30)	2 (20)
	Taking part in the study positively affected activities^b^	2 (20)	2 (20)	5 (50)
**Taking part in the study affected social contacts, n (%)**				
	Positively	1 (10)	2 (20)	7 (70)
	Negatively	0 (0)	0 (0)	10 (100)
	Satisfaction with contact with trained psychologist^b^	9 (100)	0 (0)	0 (0)
	Participant felt understood by trained psychologist^b^	9 (100)	0 (0)	0 (0)
	Self-reported level of understanding of instructions provided by trained psychologist^b^	9 (100)	0 (0)	0 (0)
**Observer-rating by trained psychologists, n (%)**				
	Compliance in session 1	7 (70)	2 (20)	1 (10)
	Compliance in session 2	7 (70)	3 (30)	0 (0)
	Compliance in session 3	6 (60)	2 (20)	2 (20)
**EMIcompass app usability, n (%)**				
	Readability of text on screen	10 (100)	0 (0)	0 (0)
	Difficulties in operating the app	0 (0)	0 (0)	10 (100)
	Clarity of instructions given on screen	10 (100)	0 (0)	0 (0)
	Difficulties understanding used items	0 (0)	2 (20)	8 (80)
**EMIcompass app perceived as burdensome, n (%)**				
	In terms of the number of signals per day	0 (0)	3 (30)	7 (70)
	In terms of the number of items asked per signal	0 (0)	1 (10)	9 (90)
	In terms of the signal sound	1 (10)	1 (10)	8 (80)
	Technical problems	0 (0)	1 (10)	9 (90)

^a^Items were rated on a 7-point Likert scale ranging from not at all (rating of 1) to moderate (rating of 4) and very (rating of 7). Trained psychologists noted the answers. The answers were grouped into three categories of not (rating of 3 or lower), moderate (rating of 4 or 5), and very (rating of 6 or 7) for the sake of interpretability (given small numbers in each cell).

^b^Missing value for 1 participant.

### Initial Therapeutic Effects

#### Stress Sensitivity, Negative and Positive Affect, and Psychotic Experiences in Daily Life

The findings on the initial therapeutic effects of the EMIcompass intervention on stress sensitivity are provided in Table 4. We found preliminary evidence that participants experienced less intense negative affect in response to event-related and activity-related stress at postintervention and in response to overall, event-related, activity-related, and social stress at follow-up than at baseline, as indicated by statistically significant two-way interaction effects for stress×time point. Furthermore, participants reported less intense psychotic experiences in response to minor stressors in daily life (ie, overall and specific types of stressors) at postintervention and follow-up than at baseline.

**Table 4:** Initial therapeutic effects of EMIcompass on stress sensitivity in daily life.

Outcome				Postintervention versus baseline				Follow-up versus baseline				Follow-up versus postintervention					Likelihood ratio test for interaction^a^		
				Adjusted β^b^ (95% CI)		*P* value		Adjusted β (95% CI)		*P* value		Adjusted β (95% CI)		*P* value		Chi-square (*df*)			PFWE^c^
**Negative affect**																			
	**Stress**																		
		Overall	−0.12 (−0.27 to 0.03)		.11		−0.51 (−0.63 to −0.40)		<.001		−0.39 (−0.55 to −0.23)		<.001		72.6 (2)			<.001	
		Event-related	−0.41 (−0.56 to −0.25)		<.001		−0.39 (−0.51 to −0.27)		<.001		0.02 (−0.14 to 0.18)		.83		51.6 (2)			<.001	
		Activity-related	−0.25 (−0.40 to −0.09)		.002		−0.35 (−0.47 to −0.23)		<.001		−0.10 (−0.27 to 0.06)		.22		32.5 (2)			<.001	
		Social	0.05 (−0.10 to 0.20)		.50		−0.41 (−0.53 to −0.28)		<.001		−0.46 (−0.62 to −0.29)		<.001		47.6 (2)			<.001	
**Psychotic experiences**																			
	**Stress**																		
		Overall	−0.15 (−0.25 to −0.04)		.005		−0.28 (−0.36 to −0.20)		<.001		−0.14 (−0.25 to −0.03)		.01		48.7 (2)			<.001	
		Event-related	−0.29 (−0.39 to −0.19)		<.001		−0.19 (−0.27 to −0.11)		<.001		0.10 (−0.01 to 0.20)		.08		40.6 (2)			<.001	
		Activity-related	−0.25 (−0.35 to −0.14)		<.001		−0.20 (−0.28 to −0.12)		<.001		0.05 (−0.06 to 0.16)		.40		33.3 (2)			<.001	
		Social	−0.01 (−0.11 to 0.09)		.86		−0.24 (−0.32 to −0.16)		<.001		−0.23 (−0.34 to −0.12)		<.001		36.3 (2)			<.001	

^a^Likelihood ratio test for stress×time interaction after inclusion in the following model: (for y_ij_ negative affect, psychotic experiences or positive affect as outcome variable): y_ij_=β_0_+β_1_(STRESS_ij_)+β_2_(TIME_j_)+β_3_(STRESS_ij_×TIME_j_)+ε_ij_.

^b^Adjusted β: standardized regression coefficients (continuous independent variables were standardized [mean 0, SD 1] for interpreting interaction terms).

^c^PFWE: family-wise error-corrected *P* values were computed by multiplying the unadjusted *P* value by the total number of tests for each outcome (N=4) to adjust signiﬁcance levels of likelihood ratio tests for two-way interactions.

Furthermore, Table 5 shows the findings of the initial effects of EMIcompass on momentary negative affect, psychotic experiences, and positive affect. There was preliminary evidence that participants experienced less intense negative affect and psychotic experiences and more intense positive affect in daily life at postintervention and the 4-week follow-up than at baseline. There was also evidence that individuals anticipated fewer threatening events in their daily lives at postintervention and the 4-week follow-up than at baseline.

**Table 5:** Initial therapeutic effects of EMIcompass on individuals’ momentary stress, negative affect, psychotic experiences, positive affect, and threat anticipation.

	Baseline, mean (SD)	Postintervention, mean (SD)	Follow-up, mean (SD)	Postintervention versus baseline			Follow-up versus baseline	
				β (95% CI)	*P* value	β (95% CI)		*P* value
Positive affect	3.9 (1.8)	4.5 (1.5)	4.3 (1.6)	0.39 (0.16 to 0.62)	.001	0.31 (0.10 to 0.52)		.004
Negative affect	2.2 (1.3)	1.8 (1.1)	1.4 (0.7)	−0.44 (−0.59 to −0.30)	<.001	−0.59 (−0.72 to −0.46)		<.001
Psychotic experiences	1.7 (0.8)	1.4 (0.9)	1.3 (0.6)	−0.25 (−0.34 to −0.16)	<.001	−0.36 (−0.44 to −0.28)		<.001
Threat anticipation	2.7 (1.9)	2.2 (1.3)	1.6 (1.1)	−0.61 (−0.83 to −0.39)	<.001	−0.96 (−1.15 to −0.76)		<.001

#### Psychotic, Depressive, Anxiety Symptoms, and General Psychopathology

The findings on the initial therapeutic effects of EMIcompass on non-EMA outcome measures are presented in Table 6. Overall, reductions in threat anticipation, psychotic, depressive, and anxiety symptoms and general psychopathology (as indexed by the Global Severity Index) of moderate-to-large effect sizes were found at the end of the 3-week intervention period (*postintervention*) and after a 4-week follow-up period (*r*=0.30-0.65). There was initial evidence, despite the small sample size and, hence, limited statistical power, that these reductions were beyond what would be expected by chance alone for psychotic symptoms at postintervention and 4-week follow-up and, at trend level, for anxiety symptoms (postintervention, 4-week follow-up) and anticipation of a positive future self (4-week follow-up). The intervention effects on depressive symptoms and general psychopathology were also of medium-to-large effect size but fell short of statistical significance. Reductions in threat anticipation (self or other) were only of small-to-moderate effect size and did not reach conventional levels of statistical significance.

**Table 6:** Initial therapeutic effects of EMIcompass intervention on psychotic, depressive, and anxiety symptoms, general psychopathology, and threat anticipation.

			Scores, median (range)						Paired Wilcoxon signed-rank test (N=10)										
			Baseline		Postintervention		Follow-up		Postintervention versus baseline				Follow-up versus baseline				Follow-up versus postintervention		
									z		Effect size (*r*)^a^		z		Effect size (*r*)^a^		z		Effect size (*r*)^a^
**BSI^b^**																			
	Global Severity Index	81 (22-146)		68.5 (5-158)		51 (7-142)		−1.02		−0.32		−1.17		−0.37		−1.53		−0.48	
	Depression	13.5 (1-23)		12 (0-23)		7 (1-21)		−1.02		−0.33		−1.03		−0.33		−1.38		−0.44	
	Anxiety	11.5 (4-16)		9.5 (0-17)		7 (2-14)		−1.74		−0.55^c^		−1.79		−0.57^c^		−0.82		−0.26	
**GPTS^d^**																			
	Total score	41 (32-73)		46.5 (32-83)		38 (32-70)		1.94		0.61^e^		−1.74		−0.55^c^		−2.50		−0.79^e^	
**Prodromal questionnaire**																			
	Total score	5 (1-10)		5 (0-9)		2 (0-10)		−1.32		−0.42		−2.05		−0.65^e^		−1.34		−0.42	
**TAM^f^**																			
	Future self (positive)	26.5 (17-37)		27 (16-37)		33 (7-42)		0.41		0.13		1.89		0.60^c^		1.79		0.57^c^	
	Future self (threatening)	15.5 (11-25)		16.5 (7-24)		13 (7-34)		−0.46		−0.15		−1.28		−0.40		−0.52		−0.16	
	Future others (positive)	31.5 (19-45)		31 (27-42)		33.5 (22-44)		0.21		0.07		1.33		0.42		1.74		0.55^c^	
	Future others (threatening)	15.5 (7-37)		14 (8-36)		13.5 (7-32)		−0.78		−0.25		−0.77		−0.24		−0.21		−0.07	

^a^Effect size estimates are based on *r* described by Rosenthal and DiMatteo [102] using the following formula: r=Z/√number of pairs.

^b^BSI: Brief Symptom Inventory.

^c^*P*<.10.

^d^GPTS: Green et al, Paranoid Thoughts Scale.

^e^*P*<.05*.*

^f^TAM: threat anticipation measure.

## Discussion

### Principal Findings

The findings of this uncontrolled phase 1 pilot study suggest initial results on the feasibility, safety, and preliminary therapeutic effects of a compassion-focused ecological momentary transdiagnostic intervention designed to improve emotional resilience to stress (*EMIcompass*) in help-seeking youth with psychotic, depressive, or anxiety symptoms. First, individuals were satisfied with face-to-face and app-based intervention components, interference with daily activities was low, and observer-rated compliance with the treatment was high. The indicators of app usability were satisfactory. In addition, no adverse effects were observed. Second, there was preliminary evidence of decreased stress sensitivity, negative affect, and psychotic experiences and increased positive affect in daily life at the end of the 3-week intervention period (*postintervention*) and after a 4-week follow-up period (*follow-up*) as compared with baseline. Third, there was initial evidence, despite the small sample size and limited statistical power, of reductions in threat anticipation, psychotic, anxiety, and depressive symptoms of medium-to-large effect size (*r*=0.30-0.65). Overall, this reflects promising preliminary evidence of clinical feasibility and safety of the EMIcompass intervention in help-seeking youth and some evidence on initial therapeutic effects. However, findings on clinical outcomes should be interpreted with caution, considering the small sample size of this pilot study.

### Strengths and Limitations

The strength of this study is that the principles of CFIs were, for the first time, translated into an EMI administered through an mHealth app as a new avenue for real-world and real-time prevention and intervention in youth. Furthermore, EMIcompass transforms evidence on putative underlying mechanisms into an intervention that directly targets these mechanisms in daily life and hence is translational. However, there are a number of limitations that must be considered when interpreting our findings. First, in line with state-of-the-art guidance on developing and evaluating complex interventions [103], mHealth interventions in particular [104], the sample size (N=10) of this pilot study was selected to be small. Thus, the primary focus of this study was to investigate feasibility and safety and estimate the effect size of initial therapeutic effects rather than statistical significance to provide the basis for a feasibility RCT [105]. Nonetheless, while considering the low statistical power and limitations associated with a small sample size, we found preliminary evidence (in terms of statistical significance) on the effects of the EMIcompass intervention on stress sensitivity. These are promising findings, as stress sensitivity is the primary target of this emotion regulation–focused intervention. Second, data on feasibility and acceptability were assessed together with or by a trained psychologist and not an independent person. Thus, we cannot rule out biases and underreporting of unhelpful experiences. Third, we used a modified version of an established debriefing scale already used for a decade in EMA studies and, more recently, in other EMIs [34,35] to assess satisfaction, engagement, and other domains of feasibility. However, the convergent validity of this measure with other established measures (eg, Mobile App Rating Scale) and other psychometric properties remain to be established. Fourth, because of the absence of a waiting list or active control group, we cannot rule out that there may be no additive therapeutic effects of the EMIcompass intervention to the therapeutic effects of the face-to-face sessions with the trained psychologists or other therapeutic interventions participants received during the intervention period in the form of treatment as usual. However, the primary aim of this pragmatic phase 1 pilot study was to provide the basis for a feasibility RCT by investigating feasibility and safety, generating initial effect sizes. Further examination of the efficacy of EMIcompass intervention is urgently warranted. Fifth, most participants were women, and half of the participants had depression, which may limit the generalizability of findings, as selection bias may have operated on our sampling procedure. Sixth, after written informed consent was obtained and baseline assessments were completed, 5 individuals decided not to participate in the study. The reasons for exclusion were not assessed, which limited our findings on feasibility. Finally, the complex nature of the investigated constructs, sample size, and study design exclude any form of causal inference.

### Ideas for Future Work

The EMIcompass intervention aimed to augment current treatment options for young individuals seeking help for mental health problems. Most individuals reported being satisfied with the intervention. Although the small sample size has to be considered when interpreting findings, the preliminary therapeutic effects on candidate psychological mechanisms, including stress sensitivity and other psychopathological outcomes, were promising. Importantly, no adverse effects have been reported, and participating in the study did not hinder individuals in their daily activities. Thus, overall, findings on feasibility, safety, and initial therapeutic effects may be considered encouraging.

This is one of the first studies to develop and pilot an EMI that incorporates an adaptive and context-dependent delivery scheme of intervention components in youth with mental health problems. The *interactive tasks* were triggered in approximately 1 out of 3 of all EMA assessments when individuals experienced elevated levels of negative affect (eg, feeling anxious, insecure, down; ie, scores higher than 4 on a 7-point Likert scale) or momentary stress. Thus, real-time data processing was successfully applied based on EMA data to determine the delivery of CFI components. This may represent not only an important step toward ecologically more valid and accessible psychological interventions in youth but also a more personalized and contextualized clinical and preventive approach. In other words, the principles of EMIs allow not only to translate intervention components targeting candidate momentary mechanisms and contexts to individuals’ daily lives but also take a personalized, adaptive approach informed by fine-grained real-time EMA data to produce sustainable change in the real world. Although a feasibility RCT is needed as a significant next step to investigate the efficacy of the intervention and feasibility as a basis for a confirmatory RCT [23,34], this pilot study of this novel EMI reflects an important stepping stone toward more personalized and accessible youth mental health care. Furthermore, in-app data analytics revealed high variability in compliance among individuals. This suggests that for some individuals, the number of signals per day was too high (ie, seven times per day on 6 consecutive days per week).

These findings hint toward potential avenues for the improvement of the EMIcompass intervention to be iteratively incorporated. First, future versions of the EMIcompass intervention may offer adaptive intervention trajectories that vary in the type of exercise depending on individual needs and preferences. Importantly, in doing so, potentially influencing factors (eg, educational level, language skills, cultural peculiarities, and subjective preferences) should be considered at an early stage of the design process and considered in optimizing EMIs further. Coproduction with young service users is essential during these developmental processes [106]. Second, sustained engagement in using digital tools remains a significant challenge [107], which may be addressed through the use of gamification elements, especially in youth [108,109]. However, in this study, the burden associated with app usage was low, and problems with engagement have mainly been reported for stand-alone mHealth apps without components of blended care [110]. Third, in working toward more personalized mHealth apps, more sophisticated methods may be used to inform the timing and context of when intervention components are offered (eg, by using mobile sensing data). A broader range of intervention components delivered for a longer intervention period may help enhance the effects of EMIcompass further and achieve sustainable change in individuals’ daily lives. Fourth, the type of intervention components may be personalized further by assessing the effects of specific intervention components on individuals’ mental health at the individual level. Fifth, it should be further examined whether and, if so, how the therapeutic alliance can be strengthened in light of a limited number of face-to-face sessions [111]. Finally, the number of signals per day triggered by the smartphone was perceived as burdensome by some participants. Thus, future versions of the EMIcompass app may lower the number of signals per day or shorten the number of items per signal [112].

### Conclusions

Evidence on feasibility and safety and preliminary evidence on the therapeutic effects of the EMIcompass intervention suggest that translating CFI components into individuals’ daily life through an EMI delivered by an mHealth app may be a promising novel, accessible, and transdiagnostic treatment approach in help-seeking youth by strengthening emotional resilience and directly targeting candidate psychological mechanisms. As an important next step, an exploratory RCT is warranted to demonstrate the feasibility and preliminary evidence of the efficacy of the EMIcompass intervention.

## Abbreviations

BSI: Brief Symptom Inventory
CBT: cognitive behavioral therapy
CFI: compassion-focused intervention
EMA: ecological momentary assessment
EMI: ecological momentary intervention
ESM: experience sampling method
mHealth: mobile health
PQ: prodromal questionnaire
RCT: randomized controlled trial
